# Receptionist rECognition and rEferral of Patients with Stroke (RECEPTS): unannounced simulated patient telephone call study in primary care

**DOI:** 10.3399/bjgp15X685621

**Published:** 2015-06-29

**Authors:** Ruth M Mellor, James P Sheppard, Elizabeth Bates, George Bouliotis, Janet Jones, Satinder Singh, John Skelton, Connie Wiskin, Richard J McManus

**Affiliations:** NHS Lanarkshire, Kirklands, Bothwell.; Department of Primary Care Health Sciences, University of Oxford, Oxford.; Primary Care Clinical Sciences;; Primary Care Clinical Research & Trials Unit, University of Birmingham, Birmingham.; Primary Care Clinical Sciences;; Northfield Health Centre (Tudor Practice), Birmingham.; Primary Care Clinical Sciences;; Primary Care Clinical Sciences;; Department of Primary Care Health Sciences, University of Oxford, Oxford.

**Keywords:** general practice, health services administration, medical receptionists, patient simulation, questionnaires, stroke

## Abstract

**Background:**

Stroke is a leading cause of morbidity and mortality. Timely recognition and referral are essential for treatment.

**Aim:**

To examine the ability of receptionists in general practices to recognise symptoms of stroke and direct patients to emergency care.

**Design and setting:**

Unannounced simulated patient telephone calls and prospective cross-sectional survey study in general practices in the Birmingham and Solihull area.

**Method:**

A total of 52 general practices participated in a total of 520 simulated telephone calls, with 183 receptionists completing questionnaires. Logistic regression analyses were used to examine likelihood of referral for immediate care by ease of vignette recognition and number of common stroke symptoms present.

**Results:**

General practice receptionists correctly referred 69% of simulated calls for immediate care. Calls classed as ‘difficult’ to recognise were less likely to be immediately referred. Compared with ‘easy’ calls: ‘difficult’ calls odds ratio (OR) 0.15, 95% confidence interval (CI) = 0.08 to 0.26; ‘moderate’ calls OR 0.55, 95% CI = 0.32 to 0.92. Similarly, calls including one or two ‘FAST’ symptoms were less likely to be referred immediately (compared with three FAST symptoms: one symptom OR 0.30, 95% CI = 0.13 to 0.72; two symptoms OR 0.35, 95% CI = 0.15 to 0.83).

**Conclusion:**

General practice receptionists refer patients with stroke for immediate care when they present with several symptoms; however, they are less likely to refer patients presenting with only one symptom or less common symptoms of stroke. Optimum management of acute stroke in primary care requires interventions that improve receptionists’ knowledge of lesser-known stroke symptoms.

## INTRODUCTION

Stroke is one of the leading causes of morbidity and mortality worldwide with an estimated 5.7 million deaths and approximately 50 million disability-adjusted life years lost every year.^1^ The burden of stroke can be reduced with thrombolysis, but this is time limited and can only be administered in a specialist setting.^2^ Thus, timely recognition and referral are essential to ensure patients with stroke receive the best possible care.

The GP is the first point of contact for between 22% and 56% of patients with acute stroke^3^ or transient ischaemic attack,^4^ but only 55–71% of these are correctly referred on to the emergency services for immediate care.^3^,^5^ Few studies have examined the mechanisms behind the referral process, although one study found that in cases where the GP answered the phone, patients were correctly referred to the emergency services.^5^ In most cases, it is the general practice receptionist who will answer a call directed to an individual’s GP and little is known about their ability to correctly recognise and refer patients with stroke.

Receptionists in this situation must determine the urgency of a patient’s condition and when (or if) an appointment should be made,^6^,^7^ but, often, their training is minimal.^8^ It is hypothesised that one source of delay to patients accessing acute stroke care is failure by receptionists to recognise symptoms of stroke and therefore to treat acute stroke patients as a medical emergency. This study aimed to examine GP receptionists’ responses to patients presenting by telephone with symptoms of acute stroke and their knowledge of stroke symptoms using unannounced simulated patients.

## METHOD

### Study design

This study examined receptionists’ responses to a series of unannounced simulated patient telephone calls. Simulated calls were performed by medical role players acting out vignettes of patients with symptoms of stroke. Individual receptionist knowledge of stroke symptoms was examined using questionnaires.

### Population

All general practices within the Birmingham and Solihull NHS Primary Care Providers (UK) area were invited to participate in the study via a combination of postal, email, and telephone invitations. Practice level consent was gained from a lead GP and the practice manager (or equivalent) at each practice. Receptionists were informed that a study was ongoing, but were not told about the nature of the simulated calls or when they would occur. All receptionists at participating practices were invited to complete a questionnaire. Practices were reimbursed a nominal amount to cover the additional work required to participate in the study. Detailed methods of the study are reported elsewhere.^9^

**How this fits in**

Many patients with acute stroke contact their GP initially, but not all are correctly referred to the emergency services. To date, little is known about general practice receptionists’ ability to correctly recognise stroke. The present study found that receptionists have good knowledge of the common symptoms of stroke. However, further consideration of a receptionist’s place at the centre of emergency primary care responses might lead to improved outcome.

### Unannounced simulated patient telephone calls

Five trained medical role players, with past experience working as simulated patients/relatives, made 10 separate calls to each participating practice. Each practice received calls from a range of role players including the same 10 different vignettes designed to include a range of stroke presentations (Box 1). Two callers specifically stated that they thought they were having a stroke, and three reported a single symptom, in keeping with stroke calls typically made to the emergency medical services (EMS).^10^ Vignettes were categorised ‘easy’, ‘moderate’ and ‘difficult’ to recognise, by an expert panel consisting of five clinicians, six receptionists (not otherwise involved in the study), and two stroke survivors. Role players performed as either the patient or a family member witnessing a patient experiencing stroke-like symptoms.

Box 1. **Details of vignettes performed in unannounced simulated patient telephone calls**

**Table:** 

**ID**	**Brain territory of stroke**	**Who**	**Vignette details^a^**	**Symptoms**	**Panel defined ease of recognition^b^**
A	Anterior	Adult/child	I think my Mum’s having a stroke: Her mouth is drooping Her speech is slurred She can’t use her right arm	Facial droop (right side), right arm weakness and speech disturbance (three FAST symptoms)	Easy
B	Anterior	Adult/child	Shall I bring my Mother in to see the doctor? She can’t use her right arm She keeps dropping things Her face is really funny (right side) She’s talking a load of rubbish	Facial droop (right side), right arm weakness and speech disturbance (three FAST symptoms)	Easy
C	Anterior	Adult/child	Do you think my Father needs to see the doctor? He’s having difficulty speaking He can’t lift his arm up	Right arm weakness and speech disturbance (two FAST symptoms)	Easy
D	Anterior	Adult/child	Can I make an appointment for my Father? His face is all lopsided (right side) He’s having trouble speaking	Facial droop (right side) and speech disturbance (two FAST symptoms)	Easy
E	Anterior	Patient	I think I need to see the doctor my daughter tells me that: My face is all droopy (left side) I keep dropping things	Facial droop (left side) and arm weakness (two FAST symptoms)	Moderate
F	Anterior	Patient	I’m not sure what to do: When I look in the mirror my reflection looks funny	Facial droop (left side) (one FAST symptom)	Moderate
G	Anterior	Adult/child	I think my Mum needs to see the doctor: Her speech is all slurred	Speech disturbance (one FAST symptom)	Moderate
H	Anterior	Patient	I think I need to see the doctor: My arm’s gone all weak	Arm weakness (one FAST symptom)	Difficult
I	Posterior	Patient	I don’t know what to do: I keep throwing up I’m feverish I have double vision	Vomiting, vertigo and visual field defect (zero FAST symptoms)	Difficult
J	Posterior	Patient	What shall I do I think I’m having a stroke? I’ve thrown up The room is spinning I have double vision	Vomiting, vertigo and visual field defect (zero FAST symptoms)	Difficult

FAST = Face Arm Speech Time test.

a If probed, symptoms were described as being ongoing and of having had a sudden onset within 2 hours of the telephone call.

b Ease of recognising symptoms was defined by an expert panel consisting of five clinicians, six receptionists (not otherwise involved in the study), and two stroke survivors.

Calls were conducted between May and October 2013, and between 10 am–12 pm and 2–4 pm. They were therefore within practice working hours, but avoiding peak time, to minimise interference with authentic patients calling the practice. To reduce the likelihood that a given simulated call might be recognised, dummy patient records were generated for each vignette and given to practice managers to upload onto practice databases prior to the initial simulated call being made. At the end of each call, receptionists were informed that it was part of a research study and that no further action should be taken. Calls were recorded and the content of each was documented on a standardised proforma by the role player conducting the simulated call.

### Questionnaires

Questionnaires were sent to all reception staff working within participating practices after completion of the simulated calls (July to October 2013). Where questionnaires were not returned within 2 weeks, a reminder was sent. The questionnaire was adapted from previous surveys used to assess general public knowledge of stroke and included questions about the demographics of the receptionist, their knowledge of stroke symptoms, and their personal experience of stroke and stroke training.^11^–^15^

The questionnaire was reviewed by non-participant receptionists to ensure that the questions were correctly understood by the intended audience. It was piloted using methods described by Eaden *et al.*^16^ The responses of 10 receptionists, three practice nurses, and five GPs were compared to ensure the questionnaire was effective at differentiating between knowledge levels.

### Data analysis

The desired sample size was 60 practices receiving 10 telephone calls each (600 calls in total), to give an expected accuracy level of ±4% based on a conservative estimate that 50% of simulated calls would be correctly immediately referred for treatment.

Data were analysed in STATA version 12. Descriptive statistics were used to describe practice and receptionist demographics, the proportion of simulated calls correctly referred for immediate care, and the number and type of stroke symptoms identified by receptionists.

Simulated call responses were categorised as immediate or delayed. Immediate responses were defined as those where a simulated patient was advised to call the EMS or transferred through to the GP immediately for triage. Delayed responses were defined as those where simulated patients were not treated as an emergency and included being advised to attend an out-of-hours service or being offered an appointment with the GP the following day. Being advised to make their own way to the emergency department was also defined as a delayed response because patients can further delay attendance if they do not recognise the urgency of the situation,^17^ and will not receive pre-hospital assessments and pre-notification by the EMS which are known to increase the likelihood of receiving emergency care (that is, thrombolysis) in acute stroke.^18^–^20^

The association between correct referral for immediate care and ease of vignette recognition or the number of FAST symptoms (Facial asymmetry, Arm weakness, and/or slurred Speech, [Time]) mentioned in the simulated call was examined using logistic regression. Simulated calls using vignettes which included the term ‘stroke’ were excluded from analyses examining association between number of FAST symptoms and referral as it was thought this might bias receptionists’ responses.

Questionnaire data from open-ended questions examining receptionist knowledge of stroke symptoms were coded as facial asymmetry, arm weakness, or slurred speech (common symptoms of anterior stroke, included in FAST^21^); vertigo/dizziness, visual disturbance or vomiting symptoms (common symptoms of posterior stroke^22^); other potentially correct symptoms (for example, numbness, confusion, or headache); incorrect symptoms; or ambiguous symptoms (where receptionists gave partial, incomplete, or non-specific answers; for example, face, speech, condition of arms for FAST).

## RESULTS

### Population characteristics

A total of 55 general practices agreed to participate in the present study, about one in five of the (233) practices in the study area. One practice subsequently withdrew from the study prior to data collection, and two further practices were unable to participate in the simulated calls as their electronic patient record system did not allow for convincing simulated records to be uploaded. The remaining 52 practices took part and were included in the final analysis. Participating general practices were representative of the local area in terms of number of GPs working in the practice, ethnic groups of practice population, and urban location, and had a range of deprivation scores, with a median score of 40.3 (Table 1). Calls took a median of 1 minute 55 seconds (interquartile range of 1 minute 22 seconds to 2 minutes 43 seconds).

**Table 1.** Demographic characteristics of participating GP practices^a^

**Characteristic**	**Practices, *n*= 52**
Practice list size, median (IQR)	4567 (4605)

Deprivation Score, based on IMD 2007, median (IQR)	40.3 (30.8)

**Ethnic groups of practice population, median (IQR)**	
White (British, Irish, Other)	82.6 (58.2)
South Asian (Indian, Pakistani, Bangladeshi, British)	8.4 (42.7)
Other	7.5 (8.6)

GPs working at the practice, median (IQR)	3 (3)

Receptionists working at the practice, median (IQR)	5 (3.3)

Calls required to be made to get 10 answered, median (IQR)	11 (3)

Answerphone message mentions stroke as a medical emergency, *n* (%)	1 (2)

a Practice demographic data correct as of 2010.

b IQR = difference between the upper and lower quartile.

### Receptionists’ responses to simulated calls

**Figure 1.**
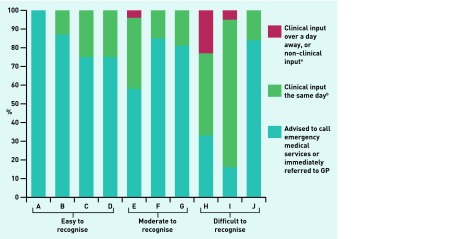
***Receptionist responses to unannounced simulated patient telephone calls by vignette. ^a^Includes: offered/given an appointment with the GP the next day, >1 day away, or asked to call back. ^b^Includes: advised to attend the emergency department; advised to attend out of hours service; advised that the GP will call back later the same day; or advised to attend the practice/walk in centre later the same day, and other responses, advising the simulated patient to call the healthcare telephone advice service and wanting to get a second opinion from the nurse.***

Of 520 simulated calls made, 69% (360/520 calls) were referred for an immediate clinical response, with most (61%, 317/520 calls) being told to call the EMS (Figure 1). ‘Difficult’ and ‘moderate’ calls were less likely to be immediately referred than ‘easy’ calls (difficult calls OR 0.15, 95% CI = 0.08 to 0.26, *P*<0.001; moderate calls OR 0.55, 95% CI = 0.32 to 0.92, *P* = 0.022). Where the term ‘stroke’ was used in a vignette reporting posterior symptoms of stroke, most responses were for immediate referral (93%; 48/52), despite the vignette being categorised as difficult to recognise (Figure 1). Calls with fewer or no FAST symptoms were less likely to be immediately referred than calls with three FAST symptoms (no FAST symptoms OR 0.03, 95% CI = 0.01 to 0.08, *P*<0.001; one FAST symptom OR 0.30, 95% CI = 0.13 to 0.72, *P* = 0.007; two FAST symptoms OR 0.35, 95% CI = 0.15 to 0.83, *P* = 0.017) (Table 2).

**Table 2.** Logistic regression demonstrating the association between referral for immediate care and the number of FAST symptoms mentioned in the simulated call^a^

**FAST symptoms, *n***	**Odds ratio**	**95% CI**	***P*-value**
3	1.00	–	–
2	0.35	0.15 to 0.83	0.017
1	0.30	0.13 to 0.72	0.007
0	0.03	0.01 to 0.08	<0.001

FAST = Face Arm Speech Time test.

a Simulated calls using vignettes A and J which included the term ‘stroke’ were excluded from the analysis as it was thought this might bias receptionists’ responses. Odds ratio > 1 indicates increased likelihood of immediate referral

### Receptionists’ knowledge of stroke symptoms

A total of 54% (183/339) of practice receptionists returned a questionnaire. Receptionists were aged 18–68 years, 86% (158/183) were female, and 73% (133/183) were white. Participants had worked as receptionists for 0–30 years and 14% (26/183) had received formal training related to stroke (Table 3).

**Table 3.** Demographic characteristics of receptionists responding to the questionnaire

	**Receptionists^a^, *n*= 183 (54%)**
Questionnaires returned per practice,^b^ median (IQR)^c^	4 (4)

**Age, years, *n* (%)**	
18–29	28 (15)
30–49	60 (33)
50–68	79 (43)

**Sex, *n* (%)**	
Female	158 (86)
Male	6 (3)

**Ethnic group, *n* (%)**	
White (British, Irish, European)	133 (73)
South Asian (Indian, Pakistani, Bangladeshi, British)	31 (17)
Mixed (white and black Caribbean/African, white and Asian)	3 (2)
Black (Caribbean, African, British)	1 (1)

**Highest level of education qualification, *n* (%)**	
Secondary education (for example, GCSEs)	110 (60)
Further education (for example, A levels)	40 (22)
Higher education (for example, university)	13 (7)
Other	7 (4)

Years worked as a GP receptionist, at any practice, median, (IQR)	7 (10)

Received formal training to recognise stroke for job, *n* (%)	26 (14)

**Experience of stroke, *n* (%)**	
Have suffered stroke	1 (1)
Have witnessed someone suffer a stroke	18 (10)
Know someone who has suffered a stroke	75 (41)
No experience of stroke	91 (50)

a Percentages do not always add up to 100 because of missing data.

b Receptionists from eight practices did not return any questionnaires.

c IQR = difference between the upper and lower quartile.

Receptionist knowledge of stroke symptoms was good: 96% (176/183) were able to name at least one symptom and 73% (133/183) could name all three FAST symptoms: facial asymmetry (89%, 162/183), slurred speech (90%, 165/183), and arm weakness (78%, 143/183). Only 29% (53/183) of receptionists reported a common symptom of posterior stroke (vomiting, visual disturbance, or vertigo), with 19% (34/183) naming visual problems and 15% (28/183) vertigo, but only 2% (4/183) identifying vomiting as a symptom. Other less specific potential stroke symptoms such as numbness, confusion, or headache, and loss of coordination were reported by 50% (91/183) of receptionists. One or more incorrect symptoms, such as chest or limb pain, were reported by 40% (74/183) of receptionists, however, and 15% (27/183) mentioned ambiguous symptoms.

## DISCUSSION

### Summary

The present study found that general practice receptionists did not always direct patients calling with potential symptoms of stroke to immediate medical advice or the EMS, particularly when the symptoms were not clear cut. They had good knowledge of common anterior circulation symptoms of stroke, and performed well when patients presented with three such symptoms (facial asymmetry, slurred speech, and arm weakness). However, presentations with fewer symptoms or those related to posterior circulation stroke were more likely to elicit a delaying response. This study used a range of scenarios, but given that only 3% of patients calling the EMS with acute stroke mention more than one symptom, there appears to be a need for new interventions to assist receptionists to recognise possible stroke. This is particularly the case in the recognition of possible posterior circulation stroke and to reinforce the need for urgent action, even when only one symptom is present.^10^ This might include training aimed at improving receptionists’ responses to stroke as part of a range of medical emergencies. Only 14% of receptionists in the current study reported receiving training related to stroke. In the UK there are no national educational structures for GP receptionists. Associated bodies can provide medical administration training; however, it is not compulsory and its scope in relation to emergency response is limited.

### Strengths and limitations

The simulated calls used here allowed examination of the actual response to patients presenting with stroke, rather than planned responses. Receptionists were informed about the nature of the study, but they were not informed that it was about stroke and calls were distributed over several months to minimise the possibility of detection. Role players were advised to be consistent in what they said, the tone of their voice, and the representation of urgency across each specific vignette. Despite this it is possible that differences in sense of urgency might have influenced receptionist response, regardless of stroke knowledge.^23^ However, this probably reflects daily practices in that the extent to which a patient is forceful, informed, or anxious could similarly influence the urgency of receptionist response. Ideally, the study would have included a sample of 60 practices, but only 52 practices had calls conducted; however, as 69% of calls were correctly immediately referred for treatment, rather than the conservative sample size calculation estimate of 50%, this smaller sample size has not affected the expected accuracy level of ±4%.

Questionnaire and simulated call data were not directly comparable as it is not known who answered the telephone during each call. The questionnaire response rate was 54% which could have introduced a response bias, but the demographics of responders were broadly in line with what was expected of participating receptionists. Questionnaires were distributed after calls were completed so it is possible that involvement in a simulated call may have influenced receptionist responses to these questionnaires.

### Comparison with existing literature

Few studies have reported how receptionists respond when patients present with symptoms of stroke.^5^,^24^ In the present study, a lower proportion (61%) of stroke calls were referred to the EMS for immediate care than in a small US study in which 71% of primary care office staff called an ambulance.^5^ Similarly, 22% of healthline operators (hospital main phone line operators) reacting to a stroke scenario recommended that patients call their GP rather than the emergency services.^25^ Differences between studies are to be expected given likely variation in call content.

Receptionists performed better than members of the public in terms of stroke symptom knowledge, with the public’s ability to name one symptom in an open-ended question ranging from 25–72%, compared with 96% in the present study.^26^ This is comparable with previous work in the US, where 95% of receptionists and 76% of healthline operators (hospital main phone line operators) could name at least one symptom of stroke.^5^,^24^

Previous general practice studies have used simulated calls.^26^–^28^ This study is novel as it is one of few studies with focus on GP receptionists and, to the authors’ knowledge, is the first to examine receptionists’ handling of emergency calls in this way.^28^ The use of human simulation as a research methodology is established, but not prolific, so this study contributes meaningfully to the evidence base for medical education. The use of simulated (‘dummy’) patient records for each caller was designed to improve the face validity of calls and reduce detection. To the authors’ knowledge, only one simulated call was recognised. The use of dummy records has not been reported elsewhere in primary care and despite initial operational difficulties was implemented successfully.^26^–^28^

Receptionists are likely to experience a wide range of emergency situations and it is important to note that increasing receptionists’ ability to recognise stroke may be only one of a number of skills needed in relation to responding to serious acute illnesses. In Australia, work is ongoing to assess how receptionists prioritise a range of situations using a decision-making tool.^29^

### Implications for research and practice

The present study found that despite generally good receptionist knowledge of stroke, there was reduced recognition of the symptoms common to posterior circulation stroke, and misinterpretation of some symptoms which were not typical of acute stroke. Previous studies have suggested that slower recognition of posterior circulation strokes in the emergency department could delay patient treatment in hospital.^30^ Indeed, the FAST test, commonly used by EMS staff in a pre-hospital setting, is less sensitive to posterior strokes, with up to 40% being classed as false negatives.^22^ This issue is likely to be compounded in primary care as patients with posterior circulation stroke are less likely to recognise their symptoms as urgent (because of the lack of media coverage highlighting these symptoms of stroke, that is, the Act FAST Campaign^21^), and therefore are more likely to initially present to non-emergency services.

The present study also found that simulated patients presenting with one or two stroke symptoms were less likely to be referred for immediate care than patients presenting with three. This perhaps reflects receptionist uncertainty about the presenting condition and increased confidence in their ability to recognise stroke where more than one symptom is present. Given the important role of receptionists in the referral of patients with stroke, interventions that improve receptionist knowledge of lesser known stroke symptoms and the need to refer patients to the EMS immediately appear relevant. Increasing receptionists’ recognition of stroke may have the unintended consequence of more stroke ‘mimic’ patients being referred for immediate care. However, recent modelling work has suggested that successfully training receptionists to refer patients for immediate care could increase thrombolysis rates by up to 16%, resulting in a greater number of patients enjoying a better quality of life after stroke and reducing resultant disability.^31^ Understanding the optimum method of achieving such changes warrants further investigation.

Receptionists have good knowledge of common stroke symptoms and are likely to refer patients presenting with these for immediate care. However, around 30% of calls made in this study were not treated as an emergency suggesting that there is still room for improvement in knowledge and behaviour. GPs and policymakers should consider the development of interventions to improve recognition of the less common symptoms of stroke, to ensure that these patients are not delayed in accessing time-dependent treatment in secondary care. Training sessions and e-learning modules could go some way to fulfilling this need.
